# Athlete students lead a healthier life than their non-athlete peers: A cross-sectional study of health behaviors, depression, and perceived health status among university students

**DOI:** 10.3389/fpsyg.2022.923667

**Published:** 2022-08-04

**Authors:** Huixuan Zhou, Yufei Zhang, Xueyan Han, Xiaotong Dai, Litian Lou, Xiao Hou, Chan Zhou, Zeting Liu, Wei Zhang

**Affiliations:** ^1^School of Sport Science, Beijing Sport University, Beijing, China; ^2^Key Laboratory of Ministry of Education for Sports and Physical Health, Beijing Sport University, Beijing, China; ^3^Peking University First Hospital, Beijing, China; ^4^School of Education, Beijing Sport University, Beijing, China; ^5^School of Sport Engineering, Beijing Sport University, Beijing, China; ^6^China National Institute for Food and Drug Control, Beijing, China

**Keywords:** health behavior, depression, athlete student, university students, health status

## Abstract

Some studies show that athlete students are more likely to engage in health-risk behaviors with negative health consequences, while others suggest that they lead a healthier life than their non-athlete peers. Given these inconsistent results, this study aims to compare health behaviors, depression, and perceived health status between athlete and non-athlete students, and explore the associations between health behaviors and health outcomes. An online questionnaire survey including Heath Habits Scale for five health-risk behaviors and five health-promoting behaviors, Patient Health Questionnaire-9 (PHQ-9), and 5-point scale for perceived health status was conducted in Beijing Sports University in March 2021. Data from 372 athlete students and 252 non-athlete students aging from 18 to 22 were included in this study. Chi-squared tests and *t*-tests were used to determine differences between athlete and non-athlete samples, and logistic regression analyses were conducted to examine the associations of health behaviors with depression and perceived health status. The significance level was *p* < 0.05. The results show that compared with non-athlete students, athlete students perform better in health habits (10.01 vs. 8.27), report lower proportion of depression (44.6% vs. 54.4%) and higher proportion of good health (77.2% vs. 55.6%). Health behaviors, such as getting adequate sleeping, participating in vigorous physical activity, overeating, and smoking, were significantly associated with health outcomes of athlete students. The findings may contribute to the better understanding of health behaviors in athlete students and warrant continued attention on mental health and health habits in this population.

## Introduction

University students are in a transitional period between adolescence and adulthood. Academic and social pressure on students may cause a series of unhealthy lifestyle with negative consequences on their physical and mental health (Mayela Nunez-Rocha et al., 2020).

Athlete students are commonly considered as one of the subpopulations facing more challenging life and more likely to engage in health-risk behaviors (Divin, 2009). Some studies have found that athlete university students are at high risk of heavy drinking and smoking compared with their non-athlete peers (Nattiv et al., 1997; Wechsler et al., 1997; Leichliter et al., 1998; Yusko et al., 2008). Moreover, some studies have shown that they are deficient in healthy diet habits (Divin, 2009; Pustivšek et al., 2019). Intentional weight gain or loss may partly explain this unhealthy pattern (Pustivšek et al., 2019). Given that they have both academic and athletic commitment and endure some pressure in the competitive environment, the depression rate of them is at approximately the same as that of their non-athlete peers (Yang et al., 2007). A number of studies have shown that the prevalence rate for depression ranges from 15.6% (Proctor and Boan-Lenzo, 2010) to 23.7% (Wolanin et al., 2016) in athlete university students.

In contrast, some studies suggest that compared with non-athlete students, athlete students lead a healthier life. Organized sport training may benefit their physical and mental health. They perform better in sleep (Edwards and Froehle, 2021), diet habits, and physical activity (Nattiv et al., 1997; Clemente et al., 2016). Moreover, the prevalence rate for depression in athlete students is lower than that among non-athlete peers (Storch et al., 2005; Armstrong and Oomen-Early, 2009; Proctor and Boan-Lenzo, 2010). In addition, an investigation in women aging 18–32 shows that athletes report better general health than non-athletes (Alamdarloo et al., 2019).

The varied investigation time and populations may have some effects on the inconsistent research findings. A majority of studies in athlete students are from the United States (Nattiv et al., 1997; Wechsler et al., 1997; Leichliter et al., 1998; Yusko et al., 2008; Divin, 2009; Proctor and Boan-Lenzo, 2010; Wolanin et al., 2016), and a few of them are from the European and middle east countries (Clemente et al., 2016; Alamdarloo et al., 2019; Pustivšek et al., 2019). The lack of evidence from Asia population and the inconsistent results of those studies warrant further research in health behaviors and health status of athlete and non-athlete students.

Additionally, previous studies examining depression in athlete students have shown that female athlete students are found to experience increased levels of depression than their male peers (Storch et al., 2005; Yang et al., 2007; Wolanin et al., 2016). However, it is unclear why there is a gender difference. Meanwhile, studies about health behaviors indicate that male athlete students perform better in diet habits than their female counterparts and have an increased risk of smoking and drinking (Nattiv et al., 1997; Yusko et al., 2008). A series of studies among university students show that health behaviors, such as sleep (Di Benedetto et al., 2020; Wickham et al., 2020), physical activity (Feng et al., 2014; Wickham et al., 2020), diet habits (Xu et al., 2016; Keck et al., 2020; Wickham et al., 2020), smoking (Velten et al., 2018; Fonseca et al., 2021; Koly et al., 2021), and drinking (Ye et al., 2016; Di Benedetto et al., 2020; Zhai et al., 2020; Fonseca et al., 2021), are associated with depression in university students. Therefore, we hypothesize that health behaviors may play a role in creating a gender gap in depression rate of athlete students.

Athletic participation in organized sport systems is getting popular in Chinese society, whether it is at youth, collegiate, and professional level. The growing interest in sport participation requires empirical research focusing on health status of athlete students in their transitional period in university. Given that health behaviors represent the leading causes of morbidity and mortality in the young-adult age group (Nattiv et al., 1997), we also need to identity whether they are at increased risks of any health behaviors with negative consequences, compared with their non-athlete peers.

Therefore, the present study compares health behaviors, depression, and perceived health status between athlete and non-athlete students in a Chinese sport university. The relationship between health outcomes and health behaviors including smoking, drinking, diet habits, sleep, and physical activity are explored in each population. In addition, we explore gender differences in health status and health behaviors and conduct multivariable regression analyses to show whether health behaviors could mediate the gender effect in health outcomes in each population. Other sociodemographic variables, including grade, family conditions, and place of hometown, which may have associations with health status of university students (Feng et al., 2014; Naser et al., 2020; Xu et al., 2020), are included in this study as covariates. The findings might help health practitioners to understand the health status of athlete university students and develop interventions targeting health behaviors associated with health outcomes.

## Materials and methods

### Participants and study design

We used a cross-sectional design through an online questionnaire survey to collect data. Athlete students were undergraduates majoring in physical education at the School of Education, Beijing Sport University. They were students with some sport skills since middle school period or earlier. They needed to complete both academic and sport training courses, and attended organized sport competitions sometimes. They were supposed to engage in physical education industry or become professional athletes according to their competition ranking and personal interests. Non-athlete students were undergraduates majoring in sport science at the School of Sport Science, Beijing Sport University. Their curriculum schedule was similar to that of general university students, which mainly consisted of academic courses.

Freshmen to seniors (traditionally aging from 18 to 22) from those two schools were invited to take part in this survey. We asked the counselors or class monitors to provide QR code for online questionnaire in the group chat of each class through WeChat, a commonly used social media application in China, and sent reminders for a total of three times in March 2021. Students completed the questionnaire anonymously and of their own free will.

The questionnaire included demographic information and measures for health behaviors, depression, and perceived health status. Missing values were avoided by the setting of required field for each question in the online survey.

The study protocol was approved by the Ethics Committee of Sport Science Experiment, Beijing Sport University (2020046H). Informed consent was provided *via* the homepage of online survey and was obtained when participants completed the survey.

### Measures

#### Health behaviors

Health Habits Scale with minor revisions according to Chinese diet habits, was used to measure 10 health behaviors with five levels of frequency: every day, several times a week, several times a month, several times a year and never (Williams et al., 1991). The value range of five health-risk behaviors, that is, smoking, alcohol drinking, overeating, eating foods high in fat and calories and eating foods high in salt, was −4 to 0 point. The higher frequency, the lower score. The value range of five health-promoting behaviors, that is, brushing teeth twice or more times a day, getting adequate sleep, drinking 800 ml or more of water a day, participating in vigorous physical activity and doing stretching exercises, was 0–4 points. The higher frequency, the higher score. The total score of the scale was −20 to 20 points.

#### Depression

Depression was measured using the Patient Health Questionnaire-9 (PHQ-9). The responses were divided into four levels according to the frequency of symptoms in the past 2 weeks: never = 0, several days = 1, more than half of the time = 2, and almost every day = 3. The total score of the scale was 0–27 points. Those scoring 5 and above had depressive symptoms. The symptoms can be divided into mild depression (5–9 points), moderate depression (10 to 14 points), and moderate to severe depression (15 to 27 points; Spitzer et al., 1999). The Cronbach’s alpha value of these 9 items was 0.88, which was larger than 0.70 and showed a good reliability in this survey (Streiner et al., 2015).

#### Perceived health status

Participant indicated their perceived health status by checking one of the following categories: very good, good, regular, poor, very poor.

### Statistical analyses

The data were analyzed using the Statistical Package of the Social Sciences (SPSS version 20; IBM Inc., Armonk, NY, United States). The demographic information, depression and perceived health status were taken as categorical variables and presented by frequency. Health habit scores were taken as continuous variables and described by mean and standard deviation (SD). The chi-squared tests (Bonferroni method) and two-tailed, between-subject *t*-tests were performed to determine differences between athlete and non-athlete samples, as well as between male and female groups inside each sample. With athlete students and non-athlete students as the analytic samples, respectively, forward stepwise logistic regression analyses were conducted to examine the association of demographics and behaviors with dependent variables, depression (presence vs. absence) and perceived health status (very poor to regular vs. good/very good). Odds ratios (ORs) were presented. The significance level was *p* < 0.05.

## Results

### Basic characteristics

A total of 624 participants completed the questionnaire, which consisted of 372 athlete students and 252 non-athlete students. The response rate was 25.0% (372 out of 1,488) among athlete students and 72.0% (252 out of 350) among non-athlete students. All collected questionnaires were involved in analysis. The basic information of the participants is shown in Table 1, stratified by the athletic identity.

**Table 1:** Characteristics of athlete and non-athlete students, *n* (%).

Variable	Athlete (*n* = 372)	Non-athlete (*n* = 252)	*p*
**Gender**			<0.001
Male	277 (74.5)	86 (34.1)	
Female	95 (25.5)	137 (65.9)	
**Grade**			<0.001
Freschman^a^	167 (44.9)	80 (31.7)	
Sophomore^a^	141 (37.9)	71 (28.2)	
Junior^a^	53 (14.2)	72 (28.6)	
Senior^a^	10 (2.7)	29 (11.5)	
**Place of hometown**			<0.001
Rural	217 (58.3)	61 (24.2)	
Urban	155 (41.7)	191 (75.8)	
**Only child**			<0.001
Yes	119 (32.0)	150 (59.5)	
No	253 (68.0)	102 (40.5)	
**Monthly family income**			<0.001
Below 3,000 CNY^a^	89 (23.9)	22 (8.7)	
3,000–5,999 CNY	119 (32.0)	76 (30.2)	
6,000–8,999 CNY	74 (19.9)	57 (22.6)	
Above 9,000 CNY^a^	90 (24.2)	97 (38.5)	
**Parents’ marital status**			0.160
Married	343 (92.2)	224 (88.9)	
Divorce	29 (7.8)	28 (11.1)	

a Significant difference between subgroups.

Comparing with non-athlete students, the proportions of athlete students of female gender, being the only child in his or her family, from urban areas, and from a family with monthly income above 9,000 CNY were significantly lower, the proportion of athlete students from a family with monthly income below 3,000 CNY was significantly higher.

### Health behaviors

The mean and SD of health behaviors of each group can be seen in Table 2.

**Table 2:** Health habit scores.

Health behaviors	Athlete			Non-athlete			Significant differences
	Male	Female	Total	Male	Female	Total	
	*n* = 277	*n* = 95	*n* = 372	*n* = 86	*n* = 166	*n* = 252	
**Brushing teeth twice or more a day**							
Mean	3.64	3.76	3.67	3.52	3.70	3.64	
SD	0.88	0.78	0.86	0.99	0.86	0.91	
**Getting adequate sleep**							
Mean	3.43	3.38	3.42	3.29	3.38	3.35	
SD	0.69	0.64	0.68	0.84	0.62	0.70	
**Drinking 800 ml or more of water a day**							A**, B***, C*
Mean	3.56	3.22	3.47	3.48	3.17	3.27	
SD	0.72	0.76	0.75	0.85	0.99	0.95	
**Smoking**							A***, B***, C**
Mean	−0.69	−0.12	−0.54	−0.33	−0.07	−0.15	
SD	1.35	0.52	1.22	0.91	0.43	0.65	
**Alcohol drinking**							A**, B*, C***
Mean	−1.03	−0.78	−0.97	−1.05	−0.60	−0.75	
SD	0.89	0.92	0.90	0.85	0.70	0.79	
**Overeating**							
Mean	−1.48	−1.59	−1.51	−1.78	−1.56	−1.63	
SD	1.15	1.14	1.15	1.18	0.92	1.02	
**Eating foods high in fat and calories**							A*, B***
Mean	−1.97	−2.41	−2.09	−2.28	−2.26	−2.27	
SD	0.99	0.92	0.99	0.86	0.67	0.74	
**Eating foods high in salt**							A*, B**
Mean	−2.00	−2.34	−2.08	−2.34	−2.29	−2.31	
SD	1.06	0.88	1.03	1.02	0.68	0.81	
**Participating in vigorous physical activity**							A***, B**, C***
Mean	3.25	2.99	3.19	2.94	2.31	2.53	
SD	0.68	0.68	0.69	0.74	0.87	0.88	
**Doing stretching exercises**							A***, C***
Mean	3.48	3.36	3.45	3.06	2.34	2.59	
SD	0.83	0.84	0.83	1.14	1.24	1.25	
**Total health habit score**							A***
Mean	10.19	9.47	10.01	8.52	8.14	8.27	
SD	4.64	3.85	4.46	4.13	3.70	3.85	

SD, standard deviation; A, comparison between athlete and non-athlete students; B, comparison between male and female athlete students; C, comparison between male and female non-athlete students.

*p < 0.05, **p < 0.01, ***p < 0.001.

Among athlete students, the mean of total health habit score was 10.01 ± 4.46, which was significantly higher than that of non-athlete students’ (8.27 ± 3.85). The scores of athlete students in items, including water intake, eating foods high in fat and calories, eating foods high in salt, participating in vigorous physical activity, and doing stretching exercises, were significantly higher than those in non-athlete students. However, they scored significantly lower than non-athlete students in smoking and drinking, indicating that they had better work out and diet habits but had higher frequency of smoking and drinking.

Compared with male athlete students, the scores of female athlete students in smoking and drinking were significantly higher, and the scores in water intake, participating in vigorous physical activity, eating foods high in fat and calories, and eating foods high in salt were significantly lower, indicating that their frequency of smoking and drinking was relatively low, but they performed worse in work out and diet habits than male athlete students.

Among female non-athlete students, the scores in items including water intake, smoking, alcohol drinking, participating in vigorous physical activity and doing stretching exercises were significantly lower than those of male non-athlete students, indicating that their frequency of smoking and drinking was relatively low, but they performed worse in work out habits.

### Depression and perceived health status of participants

Among athlete students, the prevalence rate for depression was 44.6, 74.1% of which were mild, 17.5% were moderate, and 8.4% were moderate to severe. The depression rate of female athlete students was 55.8%, which was significantly higher than that of male athlete students 40.8%. Among non-athlete students, the prevalence rate for depression was 54.4, 76.6% of which were mild, 15.3% were moderate, and 8.0% were moderate to severe. The depression rate of athlete students was significantly lower than that of non-athlete students, see Table 3.

**Table 3:** Depression and perceived health status, *n* (%).

**Depression/perceived heath status**	**Athlete**			**Non-athlete**			**Significant differences**
	Male	Female	Total	Male	Female	Total	
	*n* = 277	*n* = 95	*n* = 372	*n* = 86	*n* = 166	*n* = 252	
**Depression**							A*, B*
Yes	113 (40.8)	53 (55.8)	166 (44.6)	40 (46.5)	97 (58.4)	137 (54.4)	
No	164 (59.2)	42 (44.2)	206 (55.4)	46 (53.5)	69 (41.6)	115 (45.6)	
**Degree of depression**							
Mild	82 (72.6)	41 (77.4)	123 (74.1)	28 (70.0)	77 (79.4)	105 (76.6)	
Moderate	21 (18.6)	8 (15.1)	29 (17.5)	7 (17.5)	14 (14.4)	21 (15.3)	
Moderate to severe	10 (8.8)	4 (7.5)	14 (8.4)	5 (12.5)	6 (6.2)	11 (8.0)	
**Perceived health status**							A***, B***, C*
Very good/good	227 (81.9)	60 (63.2)	287 (77.2)	57 (66.3)	83 (50.0)	140 (55.6)	
Very poor to regular	50 (18.1)	35 (36.8)	85 (22.8)	29 (33.7)	83 (50.0)	112 (44.4)	

A, comparison between athlete and non-athlete students; B, comparison between male and female athlete students; C, comparison between male and female non-athlete students.

*p < 0.05, ***p < 0.001.

The proportion of athlete students who reported good health status was 77.2%, which was significantly higher than that of non-athlete students 55.6%. Less proportions of female athlete and non-athlete students reported good health status, compared with their male peers, respectively, see Table 3.

### Multivariate regression analyses

Taking demographic and behavioral factors as independent variables and depression and perceived health status as dependent variables, the results of stepwise logistic regression are shown in Table 4.

**Table 4:** Association of depression and perceived health status with demographic and behavioral factors.

Independent variables	Odds ratios			
	Athlete		Non-athlete	
	Depression	Perceived good health status	Depression	Perceived good health status
**Gender (ref. male)**				
Female		0.23***		0.47**
**Only child (ref. yes)**				
No		2.56**		
**Monthly family income (ref. below 3,000 CNY)**				
3,000–5,999 CNY		1.45		
6,000–8,999 CNY		2.55*		
Above 9,000 CNY		3.85**		
**Getting sufficient sleep**	0.39***	2.83***		1.81**
**Drinking 800 ml or more of water a day**				
**Smoking**		1.52***		
**Overeating**	0.67***		0.77*	
**Participating in vigorous physical activity**	0.65*	1.67*	0.68*	

Independent variables including grade, place of hometown, parents’ marital status, brushing teeth, water intake, alcohol drinking, eating foods high in fat and calories, eating foods high in salt, and doing stretching exercises were excluded from all four models.

*p < 0.05, **p < 0.01, ***p < 0.001.

Regarding depression, athlete students with higher frequency of adequate sleep (OR = 0.39, *p* < 0.001), higher frequency of participating in vigorous physical activity (OR = 0.65, *p* < 0.05), and lower frequency of overeating (OR = 0.67, *p* < 0.001) were less likely to be detected with depression. Non-athlete students with higher frequency of participating in vigorous physical activity (OR = 0.68, *p* < 0.05) and lower frequency of overeating (OR = 0.77, *p* < 0.05) were less likely to be detected with depression.

Regarding perceived health status, female athlete students were less likely to report good health status (OR = 0.23, *p* < 0.001). Athlete students with higher frequency of sufficient sleep (OR = 2.83, *p* < 0.001), higher frequency of participating in vigorous physical activity (OR = 1.67, *p* < 0.05), and lower frequency of smoking (OR = 1.52, *p* < 0.001) were more likely to report good health status. Among non-athlete students, female students were less likely to report good health status (OR = 0.47, *p* < 0.001). Non-athlete students with higher frequency of sufficient sleep (OR = 1.81, *p* < 0.01) were more likely to report good health status.

## Discussion

### Health behaviors of athlete and non-athlete students

It was found in our study that athlete students generally performed better in health behaviors and led a more active life, compared with non-athlete students. First, athlete students ate junk foods less frequently than non-athlete students, which was similar to the results of Nattiv et al.’s study (Nattiv et al., 1997). Second, their frequency of participating in vigorous physical activity and doing stretching exercises was higher than that of non-athlete students, which are somewhat consistent with results of Clemente et al.’s study indicating that female athlete students spent more time in doing light and vigorous physical activity, compared with their non-athlete peers (Clemente et al., 2016). Consistent with most of previous studies (Nattiv et al., 1997; Yusko et al., 2008; Ludvigson, 2013; Weaver et al., 2013), it was found in our study that athlete students had higher frequency in smoking and alcohol drinking, than non-athlete students. Sport-related competitiveness may serve as an important risk factor for excessive alcohol drinking of athlete students (Weaver et al., 2013).

Furthermore, it was found that female athlete students participated in vigorous physical activity less frequently than their male peers, which was inconsistent with the results of Clemente et al.’s study. In their study, no significant difference of physical activity was found between male and female athlete students. This inconsistency indicates that gender differences in health behaviors may vary across populations. In addition, our study found that female athlete students ate junk food more frequently than their male peers, which is somewhat consistent with Nattiv et al.’s study, indicating that female athlete students were more susceptible to weight anxiety and eating disorder (Nattiv et al., 1997). Consistent with previous study (Nattiv et al., 1997; Yusko et al., 2008), this study also showed that male athlete students performed worse in smoking and drinking habits than female students. The potential explanation is that male athlete students are more likely to take smoking and drinking as a normal way to relieve their pressure (Nattiv et al., 1997).

The findings necessitate attention to alcohol and tobacco use among male athlete students, and to relatively inactive lifestyle and unhealthy diet habits of female students.

### Prevalence rate for depression and perceived health status of athlete and non-athlete students

In our study, the prevalence rate for depression of athlete students was lower than that of the non-athlete students (44.6% vs. 54.4%), and depression was found to be more prevalent in female athlete students than in male athlete students (55.8% vs. 40.8%). These results were consistent with previous studies in the United States (Yang et al., 2007; Armstrong and Oomen-Early, 2009;Proctor and Boan-Lenzo, 2010; Wolanin et al., 2016). However, the depression rate of athlete students in our study are higher than that of US athlete students ranging from 15.6% to 23.7% (Proctor and Boan-Lenzo, 2010; Wolanin et al., 2016), that of Chinese university students (28.4%; Gao et al., 2020), and that of global university students (30.6%; Ibrahim et al., 2013). A moderate to severe level of depressive symptoms was 8.4% in athlete students in our study, which was higher than that in US athlete students (6.3%; Wolanin et al., 2016). Although the investigation time and measure for depression varied from study to study, our study findings add some evidence to the concept that athlete students are not immune to depression (Wolanin et al., 2016), and female athlete students are more likely to experience depression symptoms than their male peers (Yang et al., 2007; Wolanin et al., 2016). In addition, it was found in our study that athlete students reported better health status than non-athlete students, and female students were more likely to report lower health status than their male peers. These results warrant continued attention to mental and physical health of both athlete and non-athlete students.

### Association of depression and perceived health status with health behaviors

It was found in our study that the frequency of participating in vigorous physical activity was negatively associated with the presence of depression among both athlete and non-athlete students, and positively associated with perceived health status of athlete students. High levels of physical activity have been believed associated with reduction of depression and positive self-reported health (Biddle and Asare, 2011). Moreover, higher frequency of overeating and lower frequency of getting adequate sleep were found to be associated with presence of depression among athlete students, adequate sleep was also positively associated with perceived health status of both athlete and non-athlete students, and overeating was associate with non-athlete students’ perceived heath status. In addition, smoking was found negatively associated with athlete students’ perceived health.

The results coincide with those of previous studies on university students, which suggest that health behaviors, such as physical activity (Wang, 2010; Feng et al., 2014; Wu et al., 2015; Li et al., 2020; Wickham et al., 2020; Zhai et al., 2020), diet habits (Xu et al., 2016; Keck et al., 2020; Wickham et al., 2020), sleep (Feng et al., 2014; Wu et al., 2015; Di Benedetto et al., 2020; Wickham et al., 2020; Zhai et al., 2020), and smoking (Velten et al., 2018; Fonseca et al., 2021; Koly et al., 2021), can play a role in mental and physical health of university students. Future interventions could prioritize sleep, physical activity, diet habits, and quitting cigarettes to improve the well-being of athlete and non-athlete students. Moreover, longitudinal research is required to identify causal relationships between health behaviors and health outcomes. For instance, depression may be the reason for smoking, drinking, overeating, and poor quality of sleep, and some studies suggest that sleeping may have an intermediate effect between physical activity and health outcomes (Memon et al., 2021).

Previous studies suggested that female gender may be a risk factor for depression in athlete students (Storch et al., 2005; Yang et al., 2007; Wolanin et al., 2016), as well as in non-athlete students (Alamdarloo et al., 2019). In this study, gender was not associated with depression in multivariate regression analysis, which indicated that unhealthy behaviors of female students, such as less active in physical activity and eating more junk foods than male students, may mediate the effect of gender on depression. However, the negative role of female gender in perceived health status of both athlete and non-athlete students could not be mediated by other variables, which warrant further studies on why female students are at an increased risk of perceiving poorer health status than male students.

### Limitations

This study has several limitations. First, our study used a voluntary sample from students in one sports university in Beijing. Therefore, the study results may not be generalized to athlete students nationally. Second, causal relationship between health behaviors and health outcomes cannot be drawn by this cross-sectional study design. Third, variables in our research are self-reported. Therefore, the recall and reporting bias on results cannot be avoided. For instance, objective indicators for physical health are not involved in this investigation, PHQ-9 and health habits scale only provide a snapshot of students’ depression and health behaviors, as defined by the limitations of each scale. In the future, prospective cohort studies with a large sample can be carried out to present more details of athlete students’ health behaviors (i.e., smoking and drinking in or off season) and explore causality between health behaviors and health outcomes.

## Conclusion

Compared with non-athlete university students, athlete students lead a healthier life and report a lower level of depression and a higher level of perceived health status. However, athlete students are not immune to depression and health-risk behaviors, such as smoking and alcohol drinking. The findings might help health practitioners to develop interventions targeting tobacco and alcohol control in athlete students. Health behaviors, such as adequate sleeping, participating in vigorous physical activity, overeating, and smoking, are found associated with depression or perceived health status of athlete and non-athlete students. The results warrant continued attention to mental health of both athlete and non-athlete students, and further longitudinal studies on causality between health behaviors and health outcomes of these populations.

## Data availability statement

The raw data supporting the conclusions of this article will be made available by the authors, without undue reservation.

## Author contributions

HZ, YZ, WZ, and ZL conceptualized and designed the study. XD, LL, and CZ collected and validated the data. HZ, YZ, and XH analyzed the data. HZ, ZL, and XH prepared the manuscript. All authors contributed to the article and approved the submitted version.

## Funding

This study was supported by the Fundamental Research Funds for the Central Universities, Beijing Sport University (grant number 2021QN014 and 2016RB012), and the funding from Construction of Beijing Advanced Disciplines.

## Conflict of interest

The authors declare that the research was conducted in the absence of any commercial or financial relationships that could be construed as a potential conflict of interest.

## Publisher’s note

All claims expressed in this article are solely those of the authors and do not necessarily represent those of their affiliated organizations, or those of the publisher, the editors and the reviewers. Any product that may be evaluated in this article, or claim that may be made by its manufacturer, is not guaranteed or endorsed by the publisher.
